# Autopsy at Central Park Menagerie

**Published:** 1885-04

**Authors:** H. G. Beyer

**Affiliations:** U. S. Navy


### CASE DEPARTMENT.

### I.—AUTOPSY AT CENTRAL PARK MENAGERIE.

BY DR. H. G. BEYER, U. S. NAVY.

OSTRICH, *Struthio Camelus.*—Autopsy, Jan. 27, made three days after death. At this time found the bird already skinned, with its head and upper portion of spinal cord removed ; otherwise it was not injured. The body presented a fine muscular and excellent adipose development, the *pannicalus adiposus* being in some places 1½ inches thick, showing that the bird was in a well-nourished condition when he died, and, furthermore, that he could not have been sick for any length of time, but, rather, that death must have occurred suddenly.

As it was desired to preserve the skeleton from injury as much as possible, an incision was made, beginning at the hinder extremity of the carina sterni, passing over the middle xiphoid appendix, and following the median line terminating at the symphisis pubis. This incision was supplemented by a transverse one, carried along the posterior edge of the sternum; the flaps were carefully reflected.

The omentum was now lifted up and examined, together with the distended air sacs underneath, and then cut away; the crop was dissected away from the sternum and pectoral muscles. After this had been done the whole contents of the thorax were dissected out entire from before backwards, and successively examined.

Heart weighed $1\frac{3}{4}$ lbs ; thickness of wall of right ventricle, $\frac{3}{8}$ inch; that of left ventricle, $1\frac{1}{4}$ inches ; valves perfectly transparent and normal; pericardium normal.

Lungs weighed $2\frac{1}{2}$ lbs. ; unusually adherent to posterior walls of thorax, and very friable at point of adhesion. They were very much injected throughout, a frothy, sanguinolent fluid oozing out from the incisions into its substance, the greater portion of them showing hepatization, and presenting a dark, brownish red color, harder than normal lung, and sinking in water.

Kidneys weighed $1\frac{1}{2}$ lbs., besides a certain amount of congestion, nothing abnormal about them was observed; color was a cherry brown ; capsule thin, transparent, and easily removed.

Liver weighed 3 lbs. and 5 oz., and seemed normal.

Intestine, from crop to termination in cloaca, measured 67 feet 3 inches, the duodenum being of a dark red color, very much injected, the veins standing out in prominent relief, the ileum being of a slate color, and likewise injected. The walls of the crop were $1\frac{1}{2}$ inches thick, the crop itself being completely filled with grass, half digested, and pebble-stones.

Urino-genital organs normal (male); penis presented a bone bifurcated and bent at its root.

An incision through the ventral wall of the cloaca brought to light an immense cavity, occupying the entire space between the symphisis pubis and the ventral wall of the cloaca. This cavity was surrounded by a wall of muscles and connective tissue 3-8 of an inch in thickness, and completely filled to distension with a creamy fluid measuring about a pint. This fluid, examined under the microscope, was found to be alive with micrococci in all stages of development and fission ; very few pus corpuscles could be found, and those present were filled with these micrococci, carrying on their devouring work at a rapid rate.

A stone was found in the bird's mouth about $\frac{1}{2}$ inch long in the soft part of the lower mandible, about the situation of the articular, the free end pointing towards the cavity of the mouth, the attached end being embedded in the soft parts ; the whole of it was thoroughly surrounded with connective tissue capsule, and hence had become encysted.

Cause of death seems to me clearly due to an attack of acute croupous broncho-pneumonia.

